# Experimental and Numerical Investigation into the Stability Behaviour of Cable-Stiffened Steel Columns

**DOI:** 10.3390/ma15248813

**Published:** 2022-12-09

**Authors:** Pengcheng Li, Xuxiang Zhao, Jianyue Qiu, Jianghua Qian, Zhao Zhu, Zhigang Zhang, Lei Meng

**Affiliations:** 1 School of Civil Engineering, Chongqing University, Chongqing 400045, China; 2 Key Laboratory of Building Structural Retrofitting and Underground Space Engineering (Shandong Jianzhu University), The Ministry of Education, Jinan 250101, China; 3 The Fourth Construction Co., Ltd. of China Construction Eighth Engineering Division, Qingdao 266100, China; 4 School of Civil Engineering, Ludong University, 186 Hongqi Middle Road, Yantai 264025, China

**Keywords:** cable-stiffened columns, buckling behaviour, experimental investigations, eccentric loading, numerical analysis

## Abstract

A cable-stiffened steel column (CSSC) possesses superior stability behaviour compared to ordinary compression columns. In the past, the research emphasis has focused on the behaviours of stiffened columns under axial compression; investigations into their behaviour under eccentric loading is scant. This study aims to examine the buckling behaviour of CSSCs under eccentric loading using experimental and numerical investigations. The effects of pretension in cables and eccentricity on stability behaviours were studied. According to the current investigation, it can be demonstrated that the capacities of CSSCs are higher than those of ordinary compression columns. It has also been illustrated that both the buckling loads and modes of CSSCs can be changed by changing the load eccentricity; however, the modes of ordinary columns cannot be changed. These results could be of theoretical and engineering significance in the exploration of the behaviours of cable-stiffened columns.

## 1. Introduction

**Figure 1:**
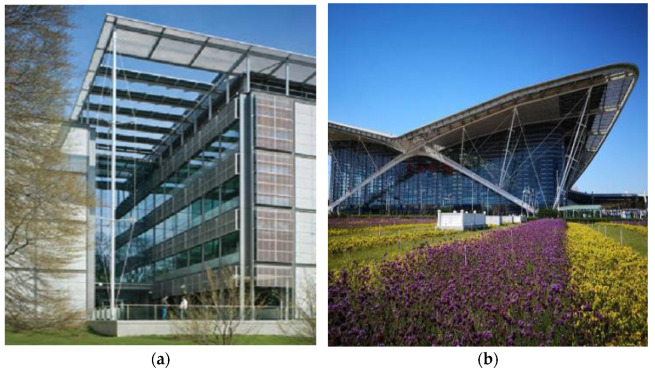
Application of CSSCs: (**a**) Princeton university [7]; (**b**) Qingdao north railway station.

In order to improve structural behaviours, some efforts are utilized extensively [1,2]. Introducing pretension into steel structures is an effective way to enhance stability behaviours. Beam string structures [3], cable-stiffened columns [4], suspended domes [5], and cable-stiffened latticed shells [6] are all typical prestressed structures that have always been used in practice. As a type of prestressed structure, a CSSC has always been used practically (see Figure 1).

Research into cable-stiffened steel columns initially concentrated on their linear buckling behaviours; this has been performed since the 1960s [8]. Then, Maunch and Felton conducted theoretical research in which it was illustrated that steel consumption can be significantly reduced by using cable-stiffened columns [9]. In the 1970s, Hafez et al. derived the relationship between initial pretension and load carrying capacities; however, the work of Hafez only focused on one-bay cable-stiffened columns [10]. By the extension of Hafez’s work, Wadee et al. derived the optimal pretensions of multi-bay CSSCs [11,12].

However, investigating the nonlinear buckling behaviour of CSSCs becomes difficult using theoretical analysis; thus, a series of finite element analyses were performed to investigate nonlinear buckling behaviours in structural engineering [13,14]. Guo et al. adopted the concept of cable-stiffened columns to propose a novel buckling restrained brace with pin-ended stays and a series of numerical analyses were performed [15]. Zschemack et al. also conducted analyses on the nonlinear buckling behaviours of CSSCs stiffened with three crossarms [16]. Chan et al. conducted numerical analyses on the capacities of CSSCs; the influencing factors of the buckling strength were investigated [17]. Martins et al. analysed the effects of steel grades on the structural behaviour of prestressed stayed columns [18]. Wu et al. conducted sensitivity analyses of buckling strength to prestressing levels, pre-cambering, and imperfections [19]. Li and Wang proposed optimisation strategies for the crossarm lengths of cable-stiffened columns and performed numerical analyses on behaviour corresponding to optimal crossarm lengths [20,21]. Hyman et al. numerically investigated the behaviour of prestressed stayed columns under eccentric loading when the main column section is circular [22]. However, the numerical results were not experimentally validated in reference [22]; in addition, the model analysed in this work was planar, which could not be found in practice.

In recent years, full-scale experiments have also been performed to examine the behaviour of CSSCs. Araujo et al. investigated the effects of different parameters on the buckling load of CSSCs; it was proved that cable-stiffened systems can improve the buckling load significantly even if the initial pretension in the cables is zero [23]. In Osofero et al., 18 cable-stiffened columns were tested; the interactive buckling phenomenon was first experimentally observed in this experiment [24]. CSSCs with lengths of 12 m were experimentally investigated by Serra et al.; the effects of column sections, cable diameters, initial pretensions, and steel grades were examined [25]. Li et al. also performed a series of experimental investigations into the stability behaviour of CSSCs with different parameters [26].

It is worth pointing out that CSSCs may be eccentrically compressed in practice; however, corresponding research on their behaviour under eccentric loads has not been systematically performed. Thus, whether the current results on CSSCs under axial compression can be used in cases when the load is eccentrically applied is unknown. Based on this background, this work aims to examine the eccentric compression behaviour of CSSCs using experimental and numerical studies. The results of this work could be of assistance for the designing of CSSCs when the compression is eccentrically loaded.

## 2. Experiment Description

**Figure 2:**
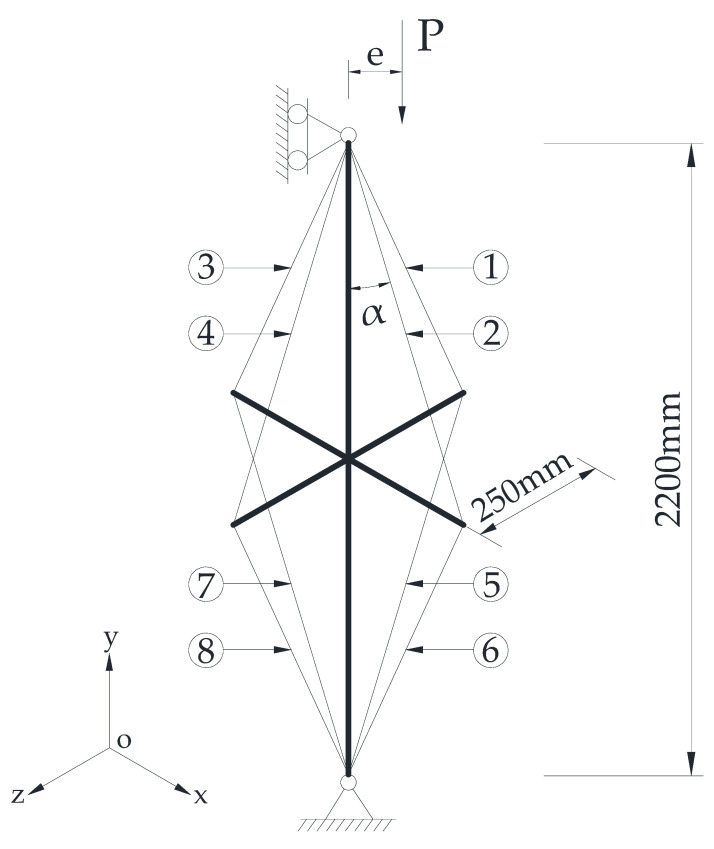
Experimental model of prestressed stayed steel column.

As mentioned above, this current work aims to investigate the buckling behaviour of CSSCs under eccentric loading. Figure 2 presents the configuration of the experimental model, in which the cables are numbered from 1 to 8. For the experimental model, the columns were pin-supported at the two ends and the crossarms were rigidly connected to the mid-span of the main column. Both the main column and crossarm were fabricated from circular steel pipes. The outer and inner nominal diameter of the column were 42 mm and 31 mm; in contrast, those of the crossarms were 20 mm and 12 mm. The nominal lengths of the crossarms were 250 mm and the diameters of the cables were 7.7 mm.

To systematically investigate the effects of eccentricity and pretension on the behaviour of CSSCs, four eccentricities varying from 0 to 30 mm and three pretension magnitudes varying from $0.5T_{opt}$ to $2T_{opt}$ were designed for the experiment. Note that $T_{opt}$ is the pretension benchmark that can be derived from geometric small deformation analysis. According to the geometric analysis, $T_{opt}$ was derived as by Hafaz, as shown in Equation (1) [8,11]:

(1) $$T_{opt}=P_{\max}^{C}C_{11}$$

In which $P_{\max}^{C}$ is the critical buckling load calculated from Equation (2) and $C_{11}$ is a coefficient that can be expressed by Equation (3):

(2) $$P_{\max}^{C}=\frac{P_{T=0}^{C}}{C_{22}}$$

(3) $$C_{11}=\frac{\cos\alpha }{2K_{c}(\frac{1}{K_{s}}+\frac{2\sin^{2}\alpha }{K_{a}}+\frac{2\cos^{2}\alpha }{K_{c}})}$$

In Equation (2), the critical load $P_{T=0}^{C}$ can be obtained from a buckling analysis in which the initial pretension is assumed to be zero, and $C_{22}$ is a coefficient that can be expressed by Equation (4):

(4) $$C_{22}=1+\frac{4\cos^{2}\alpha }{\frac{2K_{c}}{K_{s}}(1+\frac{2K_{s}\sin^{2}\alpha }{K_{a}})}$$

In Equations (3) and (4), the symbols, $K_{s}$, $K_{a}$, and $K_{c}$ represent the axial stiffness of the cables, crossarms, and columns; α is the angle between the main column and the cables.

Table 1 summarises the parameters of the steel columns that were tested in the experimental studies; the tested columns are marked by the symbol “√” in Table 1. As shown in this table, there were 8 cable stiffened columns and 4 ordinary columns tested in the comparison. The eccentricities shown in Table 1 are denoted in Figure 2.

## 3. Material Property Test

**Figure 3:**
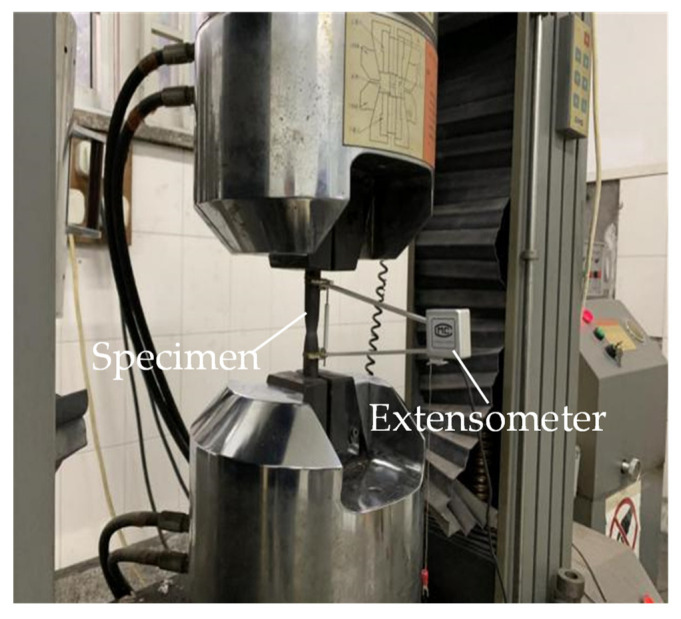
Tensile test setup.

Prior to the global buckling experiment on the columns, it was essential to conduct a material property test to obtain the actual properties of the steel and cables, as these can be used to accurately establish the numerical models in future sections. Three material specimens were cut from the main column to investigate the steel and cables’ material properties; the specimen dimensions were designed according to Metallic materials—Tensile testing—Part 1: Method of test at room temperature [27]. Similarly, there were also three specimens cut from the crossarms to perform the material test. The tensile test setup of the specimens is shown in Figure 3.

The physical properties of the main column and the crossarms are listed in Table 2. As shown in Table 2, the average buckling strength of the main column steel was 415 MPa, and that of the crossarms was 287 MPa. The ultimate tensile strength shown in Table 2 was obtained by the tensile test (see Figure 3). These magnitudes were used for the finite element analysis in Section 5.

**Figure 4:**
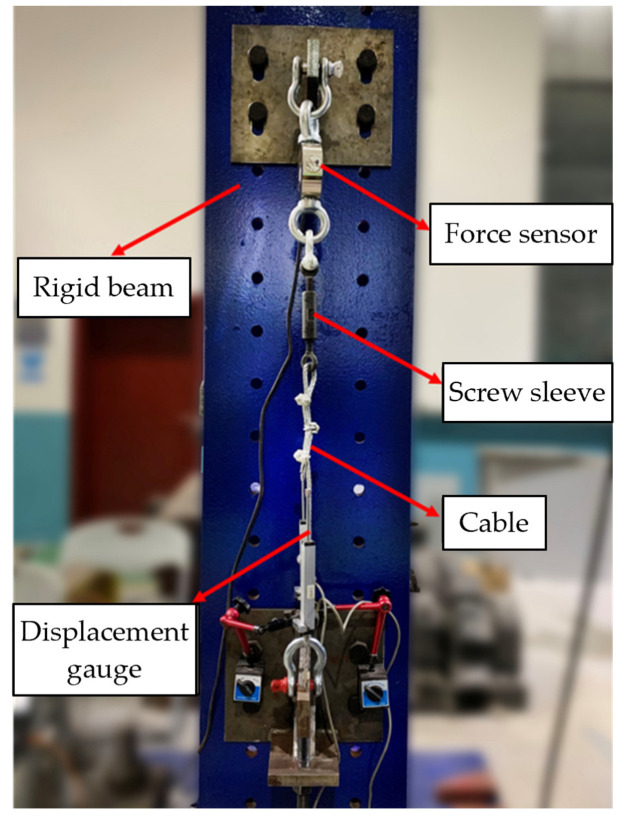
Young’s modulus examination scheme of the cable system.

For cable-stiffened columns, it is crucial to accurately introduce the pretension into cables. In the current experimental study, the pretension was introduced by a screw sleeve and measured by a force sensor. The range of the force sensor was between 0 to 5 tons, which was suitable for the experiment. In other words, the cable system was comprised of a cable, screw sleeve, and force sensor. Due to this, the screw sleeve and force sensor were also able to contribute to the stiffness of the cable systems. The stiffness of the cable systems was examined prior to the compression test, as shown in Figure 4. As shown in Figure 4, the cable system was connected to a rigid beam, and the cable system could be stretched by turning the screw. The extension of the cable system and the pretension in the cable were recorded by the displacement gauge and force sensor, respectively.

**Figure 5:**
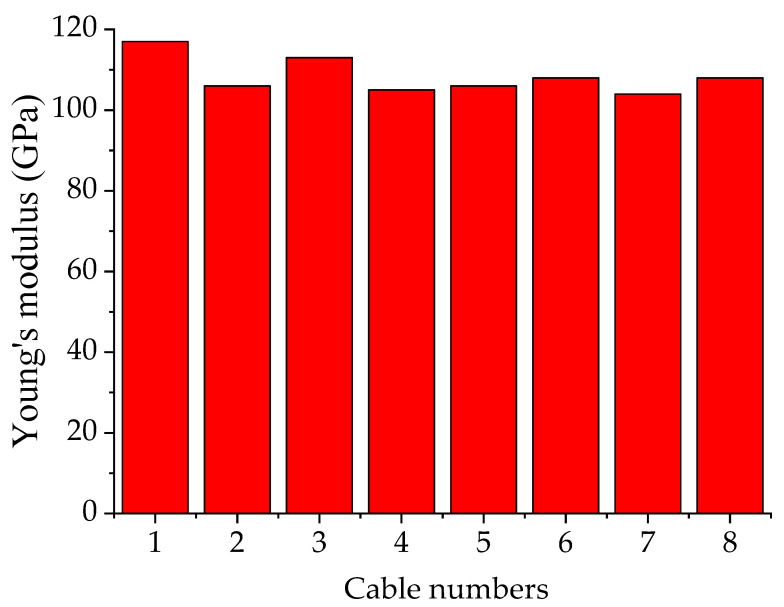
Young’s modulus of the cable systems.

As shown in Figure 2, 8 cable systems were used to connect the crossarms and main column for each CSSC. Figure 5 presents the tested Young’s modulus of the cable systems, and it was found that the Young’s modulus of the cable systems was between 100 GPa and 120 GPa. The cable numbers shown in Figure 5 are the same as those in in Figure 2. Although there were 8 cable-stiffened steel columns in the experimental investigation (see Table 1), the cable systems were repeatedly used during the experiment. In other words, the 8 cable systems were used for all the stiffened columns.

## 4. Column Compression Test

### 4.1. Test Scheme

**Figure 6:**
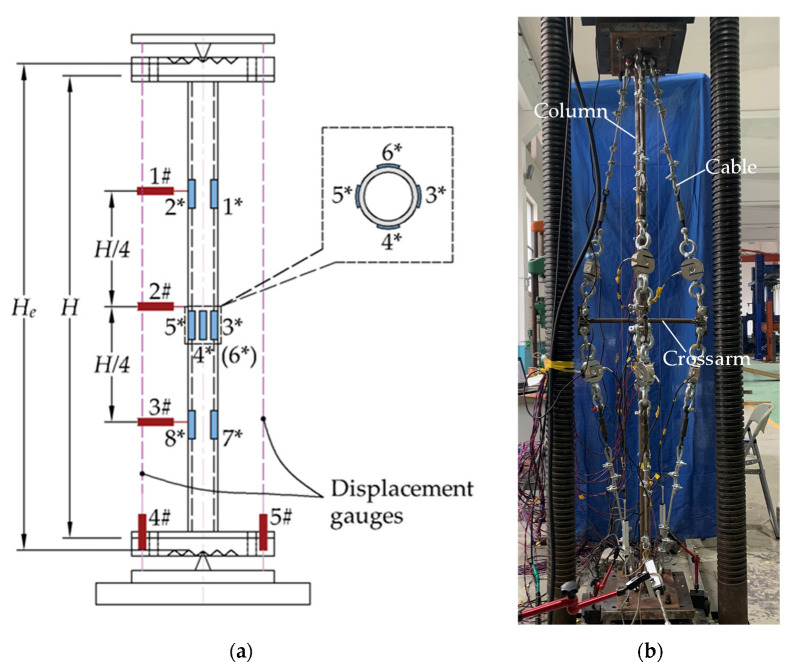
Compression column test: (**a**) Measuring point arrangement (the cables are not depicted); (**b**) Experimental scenario.

The global buckling experiments of the columns were performed using a compression testing machine. Owing to the spatial limitations of the machine, the column and crossarm lengths were designed to be 2200 mm and 250 mm. The columns were pin-supported by knife edges; the test scheme of the CSSCs is presented in Figure 6. As shown in Figure 6a, three displacement gauges numbered 1#, 2#, and 3# were set at the quadrisections of the main column—these three displacement gauges were used to record the lateral deflections that describe the buckling mode. In addition, two displacement gauges numbered 4# and 5# were used to record the axial compression of the main column. There were also 8 strain gauges numbered from 1* to 8* set at the quadrisections to record the strain of the main column. As shown in Figure 6b, there were eight cables for a CSSC. Thus, the cables were tensioned one by one in order to obtain the designed pretension.

### 4.2. Experimental Preparation

#### 4.2.1. Geometrical Configurations

The nominal outer diameter and thickness of the main column were 42 mm and 5.5 mm, and those of the crossarms were 20 mm and 4 mm. However, it must be noted that the actual dimensions were usually different from the nominal ones because of manufacturing errors. Thus, the actual dimensions had to be measured prior to the compression test. Table 3 and Table 4 list the measured dimensions of the 8 cable-stiffened columns and the 4 ordinary columns. As shown in these two tables, the actual dimensions were quite close to the nominal ones.

**Figure 7:**
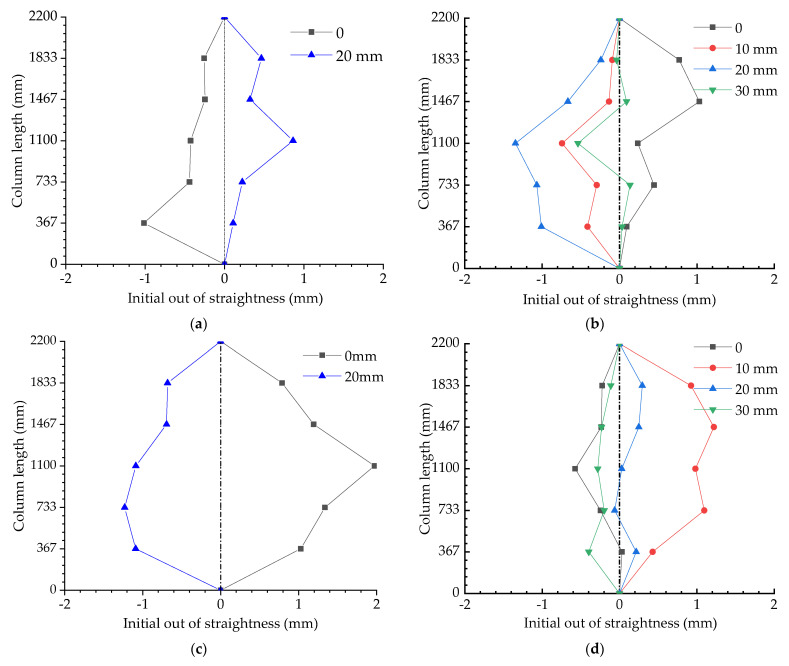
Initial imperfections of cable-stiffened and ordinary columns: (**a**) $T=0.5T_{opt}$; (**b**) $T=T_{opt}$; (**c**) $T=2T_{opt}$; (**d**) Ordinary columns.

In addition to dimension errors, the initial out-of-straightness of the columns also had to be measured. Five measured points along the column lengths were used to depict the initial configuration of the columns; the initial out-of-straightness of the cable-stiffened and ordinary columns are shown in Figure 7. The horizontal and vertical axes in Figure 7 denote the initial out-of-straightness magnitudes and column lengths, respectively. As can be seen, the maximum out-of-straightness of the main column was no more than 2 mm and L/1000 (L is the main column length).

#### 4.2.2. Initial Pretension

According to Equation (1), it is possible to calculate the initial pretension for the compression columns. Table 5 presents the designed initial pretensions calculated from Equation (1). As can be seen, the initial pretension varied from 1.15 kN to 4.58 kN.

**Figure 8:**
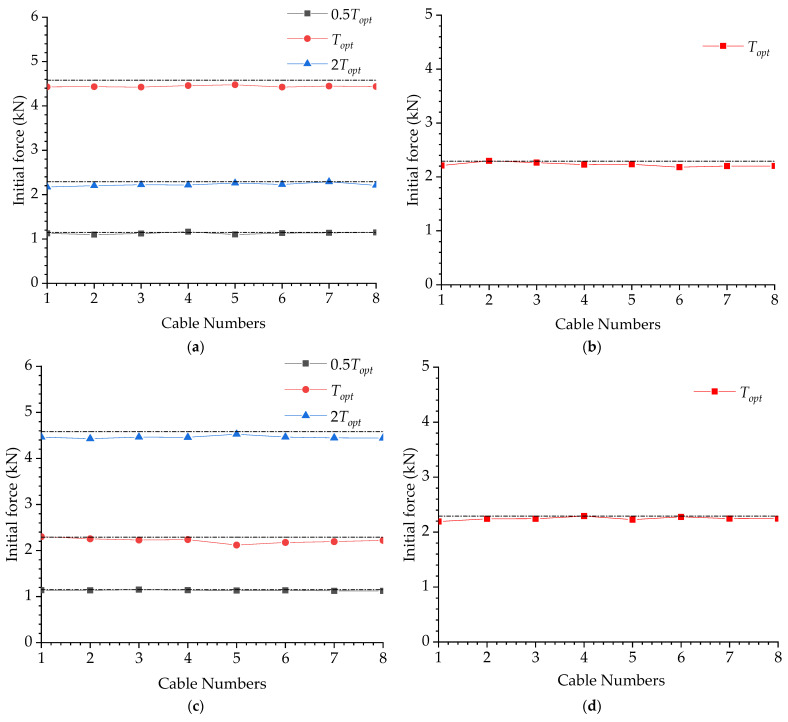
Initial pretension in the cables: (**a**) e = 0 mm; (**b**) e = 10 mm; (**c**) e = 20 mm; (**d**) e = 30 mm.

Figure 8 presents a comparison between the designed and actual pretension in cables with different eccentricities. The designed pretension is denoted by horizontal dash lines in Figure 8. Obviously, the actual pretension in cables was similar to the designed magnitudes; the maximum deviation was no more than 10%.

### 4.3. Experimental Results

#### 4.3.1. Experimental Results of Stiffened Columns with $T=T_{opt}$

The nominal outer dimeter and thickness of the main column were 42 mm and 5.5 mm, and those of the crossarms were 20 mm and 4 mm. However, it must be noted that actual dimensions usually differ from nominal dimensions because of manufacturing errors. Thus, the actual dimensions should be measured prior to compression tests. Table 3 and Table 4 list the measured dimensions of the eight cable-stiffened columns and the four ordinary columns. As shown in these two tables, the actual dimensions were quite close to the nominal ones.

(1)
e=0

**Figure 9:**
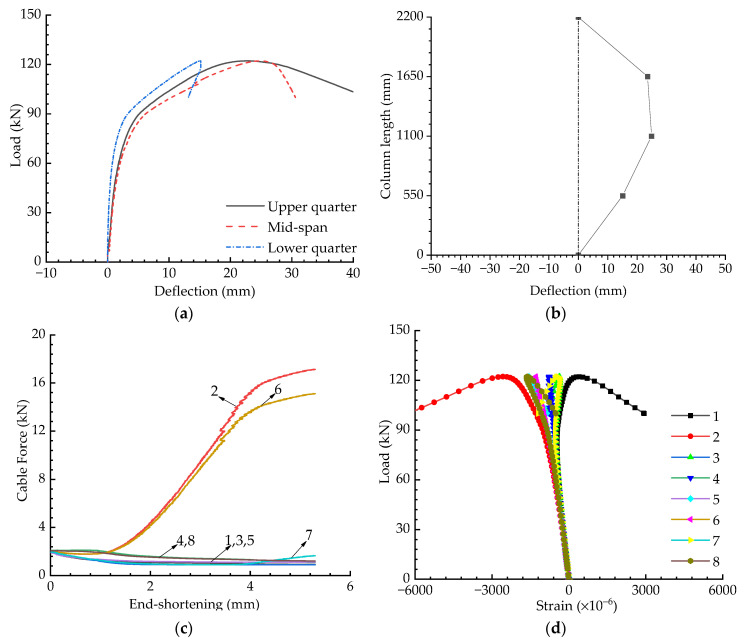
Experimental results when $T=T_{opt}$ and e=0: (**a**) Load versus deflection curves; (**b**) Buckling mode; (**c**) Pretension force versus compression curves; (**d**) Load versus strain curves.

Figure 9 presents the results for the cable-stiffened columns when the initial pretension was $T_{opt}$ and the eccentricity was zero. The load versus deflection curves are shown in Figure 9a, in which the deflections were recorded by the 1# to 3# displacement gauges (see Figure 6). The buckling mode corresponds to the instant when buckling occurs and is depicted in Figure 9b; this buckling mode was obtained from Figure 9a. The variations in pretension forces and strains are shown in Figure 9c,d—note that the cable numbers in Figure 9c are the same as those in Figure 2. As shown in Figure 9c, the pretension forces were always greater than zero, implying that all the cables were tightened during the whole process.

(2)
e=10 mm

**Figure 10:**
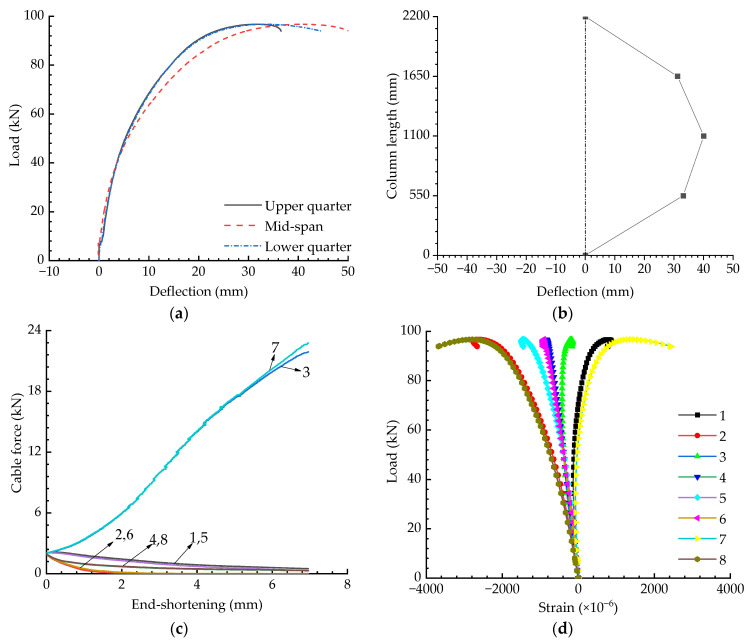
Experimental results when $T=T_{opt}$ and e=10 mm: (**a**) Load versus deflection curves; (**b**) Buckling mode; (**c**) Pretension force versus compression curves; (**d**) Load versus strain curves.

The axial compression test results are shown in Figure 9, and the buckling behaviour of the cable-stiffened steel columns under eccentric loading will be discussed in following sections. Figure 10 presents the experimental results for the cable-stiffened columns when the initial pretension was $T_{opt}$ and the eccentricity was 10 mm. Compared with the results shown in Figure 9a and Figure 10a, it could be found that the buckling strength obviously decreased when the external load changed from an axial load to an eccentric load. Differently to the axially loaded case, it could also be seen that some of the cables slacked in the eccentrically loaded case (see Figure 10c).

(3)
e=20 mm

**Figure 11:**
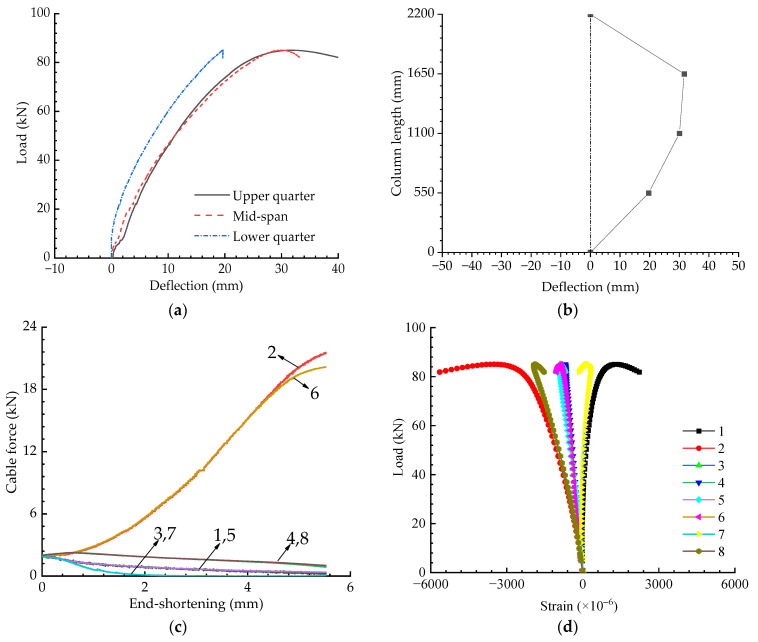
Experimental results when $T=T_{opt}$ and e=20 mm: (**a**) Load versus deflection curves; (**b**) Buckling mode; (**c**) Pretension force versus compression curves; (**d**) Load versus strain curves.

Figure 11 presents the experimental results for when the eccentricity was 20 mm with an initial pretension level of $T_{opt}$. It can be seen from Figure 11 that the buckling strength decreased in line with increasing eccentricity. Cables 3 and 7 slacked when the eccentricity was 20 mm; however, Cable 2 and 6 slacked when the eccentricity was 10 mm—this is because the columns buckled in opposite directions for these two cases.

(4)
e=30 mm

**Figure 12:**
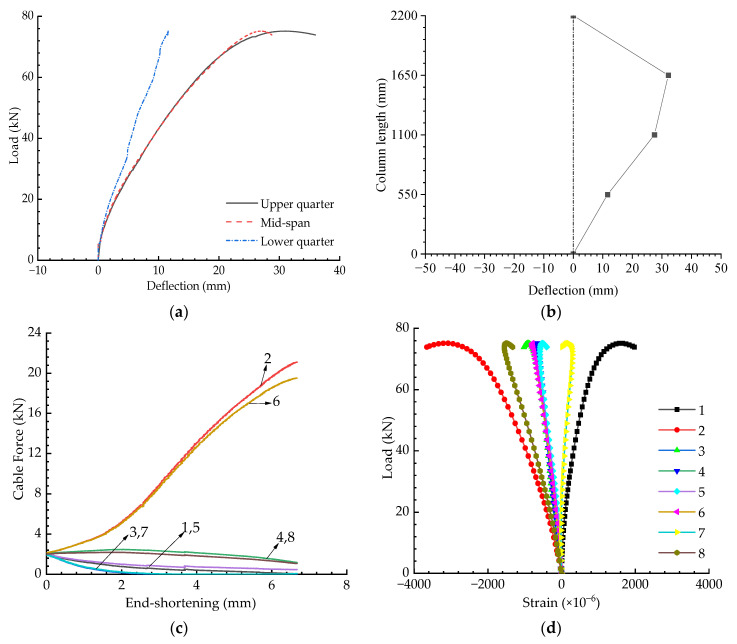
Experimental results when $T=T_{opt}$ and e=30 mm: (**a**) Load versus deflection curves; (**b**) Buckling mode; (**c**) Pretension force versus compression curves; (**d**) Load versus strain curves.

Figure 12 presents the experimental results for when the eccentricity was 30 mm and the initial pretension was $T_{opt}$. The buckling strength for this case was less than 80 kN, as shown in Figure 12a; however, the strength for the axially loaded case was around 120 kN (see Figure 9a). In other words, the buckling strength was decreased by a third when the eccentricity increased from 0 to 30 mm. As can be observed in Figure 12b, it can be found that the buckling mode changed to be asymmetric in this case. Due to this, the strains obtained from Gauges 2 and 8 or 1 and 7 were different although they were all symmetrically placed around the mid-span section.

#### 4.3.2. Experimental Results for Stiffened Columns with $T=0.5T_{opt}$

(1)
e=0

**Figure 13:**
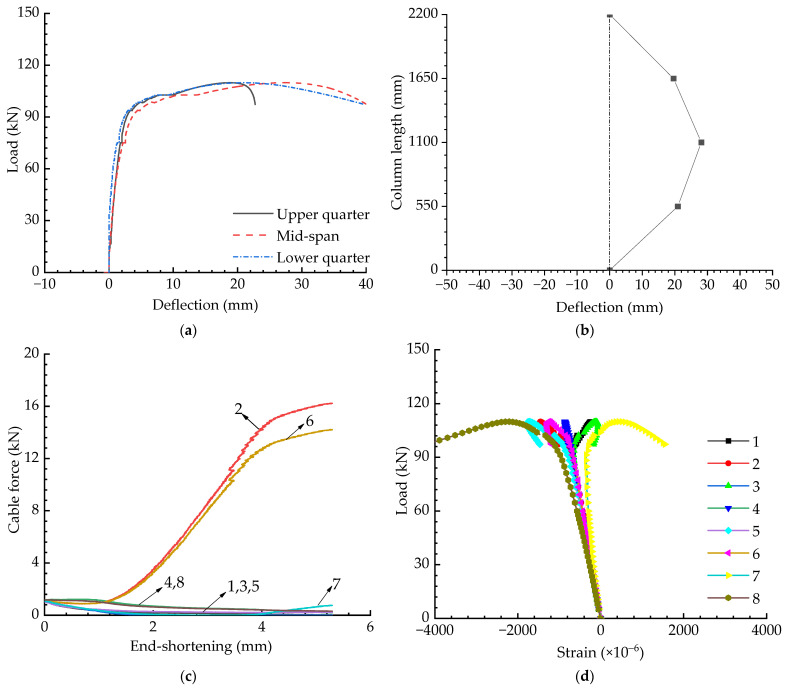
Experimental results when $T=0.5T_{opt}$ and e=0: (**a**) Load versus deflection curves; (**b**) Buckling mode; (**c**) Pretension force versus compression curves; (**d**) Load versus strain curves.

In the above section, the initial pretension in the cables was designed to be $T_{opt}$. To explore the behaviour of cable-stiffened steel columns with different initial pretension levels, the initial pretension in this section was designed to be $0.5T_{opt}$. Figure 13 presents the experimental results when the initial pretension was $0.5T_{opt}$ and the eccentricity was zero. Obviously, some cables slacked in this case (see Figure 13c); this differs from the situation when the design initial pretension was $T_{opt}$, in which all the cables were always tightened (see Figure 9c).

(2)
e=20 mm

**Figure 14:**
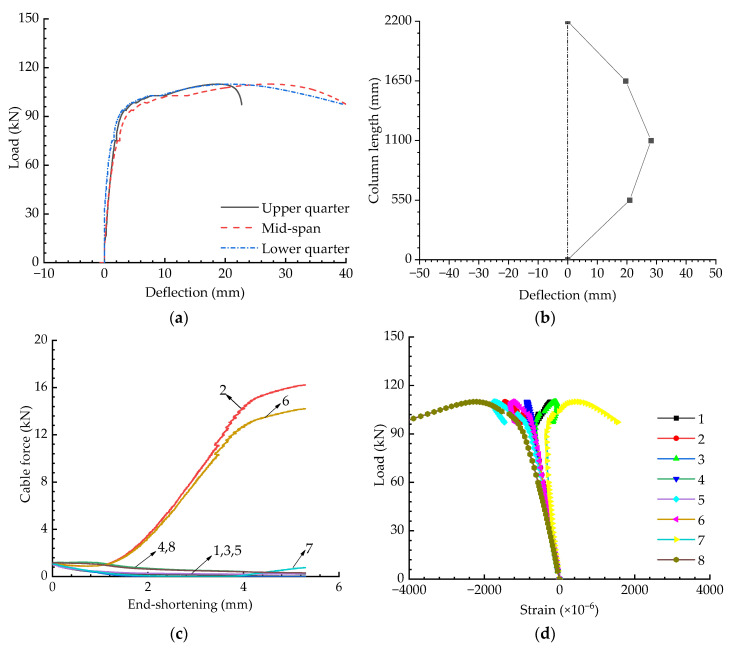
Experimental results when $T=0.5T_{opt}$ and e=20 mm: (**a**) Load versus deflection curves; (**b**) Buckling mode; (**c**) Pretension force versus compression curves; (**d**) Load versus strain curves.

Figure 14 presents the results for the cable-stiffened columns with $0.5T_{opt}$ pretension and 20 mm eccentricity. Compared to the results shown in Figure 11, it can be concluded that increasing the pretension in cables could improve buckling strength, although cable slack was observed in these two cases.

#### 4.3.3. Experimental Results for Stiffened Columns with $T=2T_{opt}$

(1)
e=0

**Figure 15:**
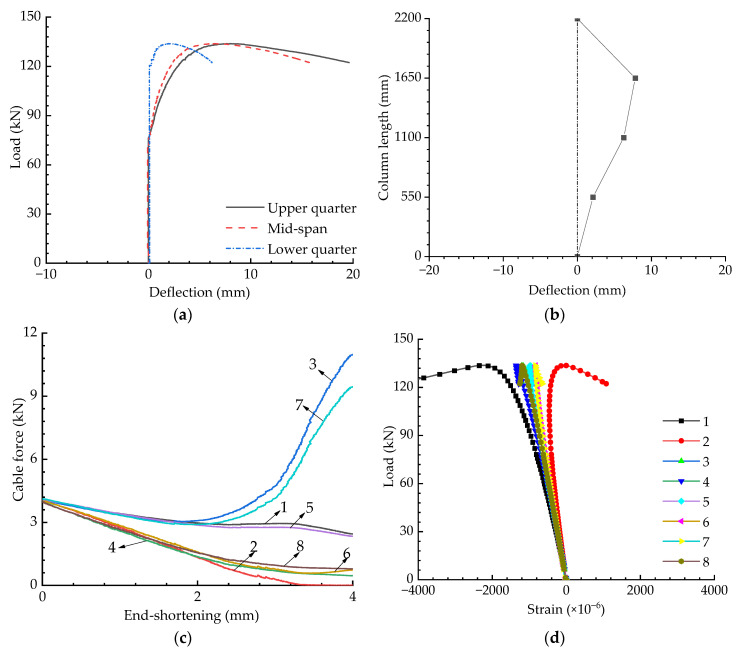
Experimental results when $T=2T_{opt}$ and e=0: (**a**) Load versus deflection curves; (**b**) Buckling mode; (**c**) Pretension force versus compression curves; (**d**) Load versus strain curves.

In this study, the initial pretension $T_{opt}$ was used as the benchmark, and this magnitude was derived from the critical buckling load. Thus, whether $T_{opt}$ was the actual initial pretension is unknown. To investigate this, the initial pretension in this section was designed to be $2T_{opt}$. Figure 15 presents the results for the cable-stiffened steel columns when $T=2T_{opt}$ and e = 0. Obviously, the buckling strength was significantly enhanced with increasing of the initial pretension from $0.5T_{opt}$ to $2T_{opt}$.

(2)
e=20 mm

**Figure 16:**
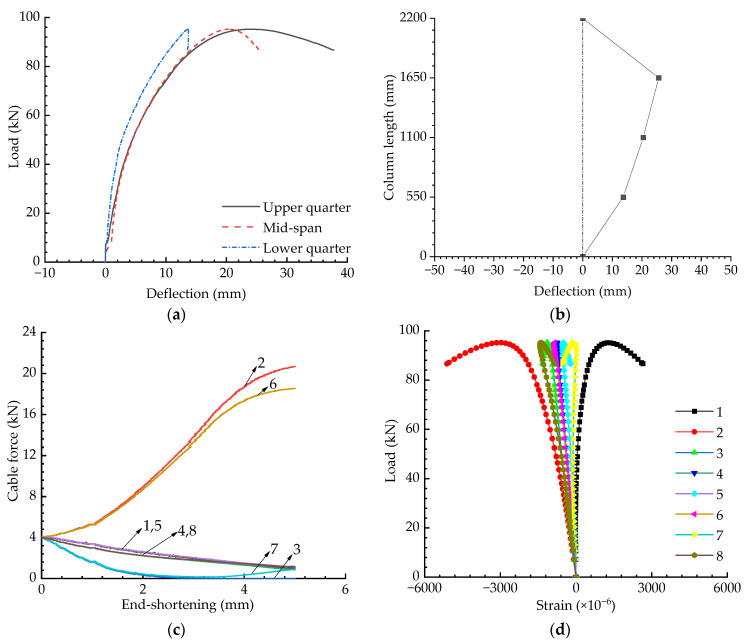
Experimental results when $T=2T_{opt}$ and e=20 mm: (**a**) Load versus deflection curves; (**b**) Buckling mode; (**c**) Pretension force versus compression curves; (**d**) Load versus strain curves.

Figure 16 presents the experimental results for the stiffened columns with an initial pretension of $2T_{opt}$ under eccentric loading (e = 20 mm). Similar to the axial-loaded case, buckling strength was also enhanced by improving the initial pretension in the cables (see Figure 11a, Figure 14a, and Figure 16a). Compared to the buckling mode shown in Figure 13b and Figure 16b, it can be illustrated that eccentric loading was able to change the mode from symmetrical to asymmetrical.

#### 4.3.4. Experimental Results for Ordinary Compression Columns

**Figure 17:**
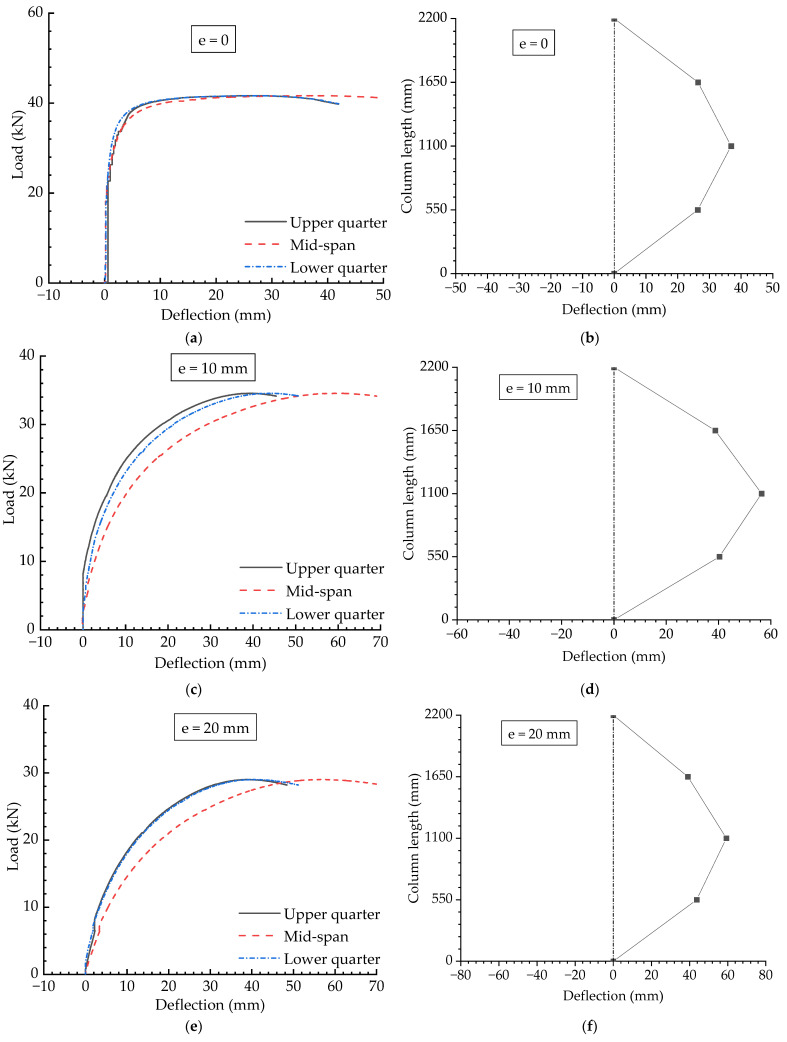

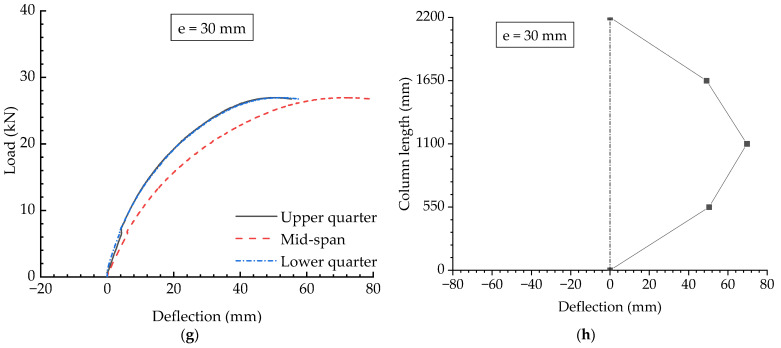
Experiment results for ordinary compression columns: (**a**) Load versus deflection curves when e=0; (**b**) Buckling mode when e=0; (**c**) Load versus deflection curves when e=10 mm; (**d**) Buckling mode when e=10 mm; (**e**) Load versus deflection curves when e=20 mm; (**f**) Buckling mode when e=20 mm; (**g**) Load versus deflection curves when e=30 mm; (**h**) Buckling mode when e=30 mm.

To investigate the effects of cable systems in improving buckling strength, ordinary compression columns without cables were also tested. Figure 17 presents the experimental results for ordinary columns with different eccentricities. It can be observed that the buckling strength decreased from around 40 kN to less than 30 kN when the eccentricity changed from 0 to 30 mm. However, the two magnitudes for cable-stiffened columns with $T_{opt}$ initial pretension were about 120 kN and 75 kN, which is more than one to two-times greater than those of the ordinary column. In addition, it can also be found that the buckling modes for the ordinary column were always symmetrical. The buckling modes of ordinary columns with different eccentricities are shown in Figure 17b,d,f,h. As can be seen, the buckling modes for these cases were all symmetrical and were not affected by the eccentricity. This characteristic is different than that of CSSCs.

## 5. Numerical and Experimental Results Comparison

### 5.1. Numerical Investigation

In addition to the experimental investigation, a numerical analysis was also performed for comparison with the experimental results. All the geometric and mechanical parameters in the numerical analysis were the same as the measured magnitudes obtained from the experiments. The numerical analysis in this section was conducted using ABAQUS. Beam elements were used to simulate the main column and crossarms; truss elements were adopted to simulate the cables.

#### 5.1.1. Cable-Stiffened Steel Columns

**Figure 18:**
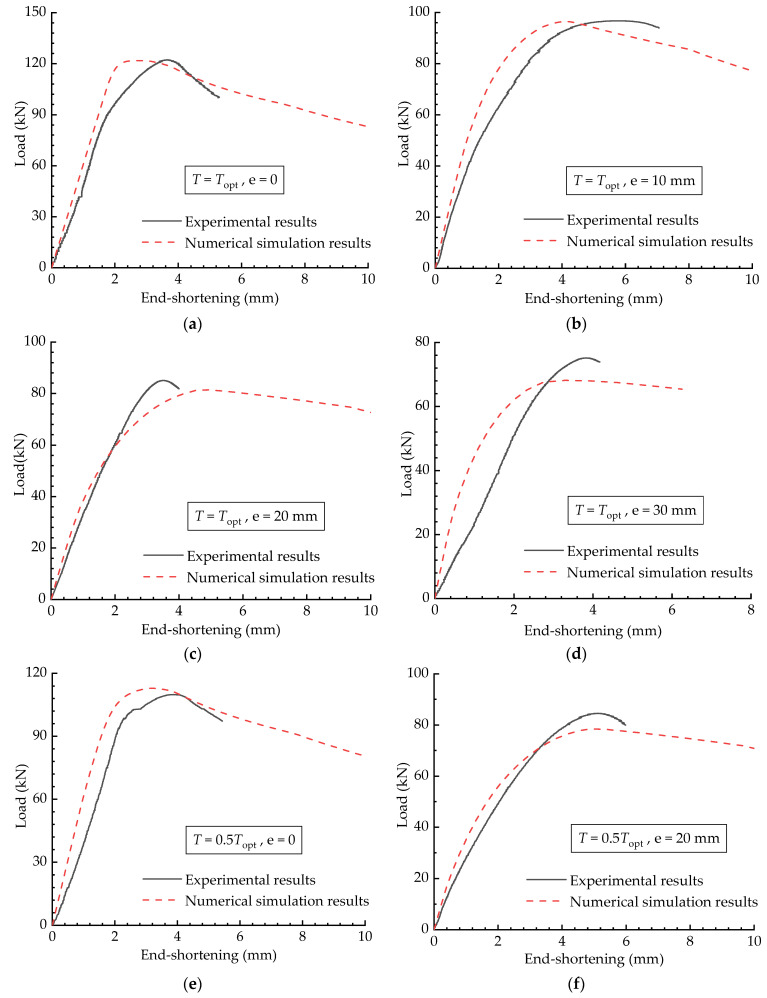

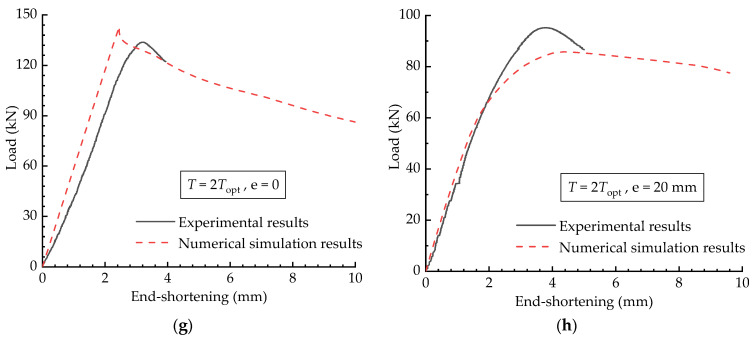
Load versus axial displacements of the CSSCs: (**a**) $T=T_{opt},\;e=0$; (**b**) $T=T_{opt},\;e=10\;\mathrm{mm}$; (**c**) $T=T_{opt},\;e=20\;\mathrm{mm}$; (**d**) $T=T_{opt},\;e=30\;\mathrm{mm}$; (**e**) $T=0.5T_{opt},\;e=0$; (**f**) $T=0.5T_{opt},\;e=20\;\mathrm{mm}$; (**g**) $T=2T_{opt},\;e=0$; (**h**) $T=2T_{opt},\;e=20\;\mathrm{mm}$.

Figure 18 presents the load versus axial displacements of CSSCs with varying initial pretension and eccentricity; the curves obtained from the numerical analysis and experimental investigation are plotted in Figure 18. Figure 18a–d shows the experimental and numerical comparisons with different eccentricities when the pretension was $T_{opt}$; as can be seen, the capacity decreased by half when the eccentricity increased from 0 to 30 mm. Similar decreases also tended to be observed in Figure 18e,f and Figure 18g,h.

#### 5.1.2. Ordinary Columns

**Figure 19:**
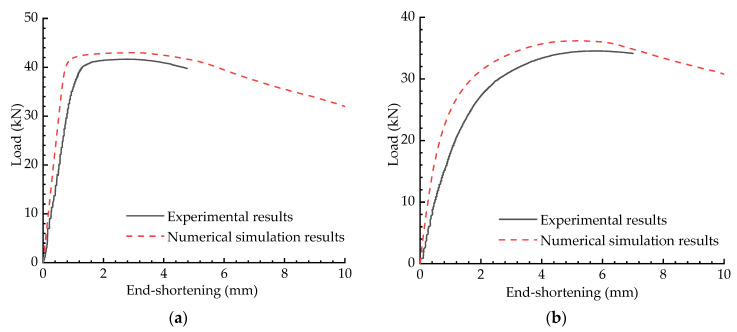

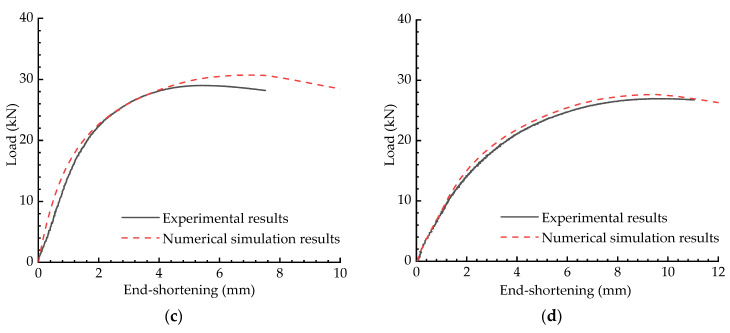
Load versus end-shortening curve comparison of the ordinary columns: (**a**) e=0; (**b**) e=10 mm; (**c**) e=20 mm; (**d**) e=30 mm.

In addition to the cable-stiffened columns, the numerical and experimental results comparison of the ordinary compression columns has also been presented (see Figure 19). According to the results shown in Figure 19, the numerical and experimental results were in good agreement with each other; it also can be noted that the capacity and initial structural stiffness were considerably decreased by increasing the eccentricity. Compared to the results shown in Figure 18, it can also be demonstrated that the capacity was only about a third of that of the CSSCs when the corresponding initial pretension was $T_{opt}$. In other words, it was much more effective to enhance the stability behaviour of the ordinary column by introducing pretensioned cables.

## 6. Conclusions

A series of experimental and numerical analyses into the behaviour of CSSCs under eccentric loads were conducted in this study. The behaviour of ordinary columns and CSSCs were compared and the influential factors for the behaviour of the CSSCs were examined. Based on the experimental and numerical studies, the below conclusions were made:

(1) The stability behaviours of the ordinary compression steel column were significantly enhanced by introducing prestressed cables; they could be improved by about three times if the initial pretension is appropriately designed.
(2) The effect of eccentricity on the buckling behaviour of CSSCs differed from that seen for the ordinary columns. For the ordinary compression columns, the buckling load was decreased from 41.65 kN to 26.94 kN when the eccentricity was increased from 0 to 30 mm, but for the CSSCs the load decreased from 122.16 kN to 75.09 kN when the initial pretension was $T_{opt}$. In contrast, the buckling mode for the ordinary columns could not be changed by the variation of eccentricity, but that of the CSSCs could be affected by the eccentricity.
(3) Initial pretension could affect the load-carrying capacities of CSSCs for both axial and eccentric loading cases; it has also been proven that the pretension level derived from the small deformation assumption does not correspond to the maximum buckling load.

According to the current work, it has been demonstrated that introducing pre-stressed cables is an efficient and lightweight way to enhance the behaviour of steel structures. However, there are many factors that could affect their structural behaviours. For CSSCs, the initial pretension should be designed and calculated carefully. Based on the numerical and experimental results of this study, an initial pretension of 2 *T_opt_* is suggested in order to obtain a higher load-carrying capacity. It must be pointed out that this current work only focuses on steel columns stiffened by single-bay crossarm systems. However, steel columns can also be stiffened with multi-bay crossarms in practice. Thus, research work on the behaviour of CSSCs with multi-bay crossarms is worthy of being performed in the future.

## Figures and Tables

**Table 1:** Structural parameters of the steel columns.

Column Types	Initial Pretensions	Eccentricities (mm)			
		0	10	20	30
Cable-stiffened columns	$0.5T_{opt}$	√	——	√	——
	$T_{opt}$	√	√	√	√
	$2T_{opt}$	√	——	√	——
Ordinary columns	No cables	√	√	√	√

**Table 2:** Physical properties of the steel.

Specimen Types	Specimen No.	Young’s Modulus (MPa)	Buckling Strength (MPa)	Ultimate Tensile Strength (MPa)
Main column	1	213	415	545
	2	209	410	550
	3	203	420	540
	Average	208	415	545
Crossarm	1	173	285	405
	2	188	290	405
	3	181	285	410
	Average	181	287	407

**Table 3:** Measured dimensions of the cable-stiffened steel columns (mm).

Pretensions	Types	Eccentricities							
		0 mm		10 mm		20 mm		30 mm	
		Column	Crossarm	Column	Crossarm	Column	Crossarm	Column	Crossarm
$0.5T_{opt}$	Outer diameters	42.31	19.96	——	——	42.31	19.87	——	——
	Thicknesses	5.59	4.00	——	——	5.52	3.93	——	——
	Lengths	2199	249	——	——	2198	250	——	——
$T_{opt}$	Outer diameters	42.22	19.81	42.39	19.86	42.34	19.99	42.24	19.89
	Thicknesses	5.50	3.95	5.46	3.91	5.68	3.96	5.54	3.88
	Lengths	2199	250	2198	251	2200	250	2199	250
$2T_{opt}$	Outer diameters	42.34	20.02	——	——	42.26	19.91	——	——
	Thicknesses	5.57	3.92	——	——	5.43	3.89	——	——
	Lengths	2199	248	——	——	2200	249	——	——

**Table 4:** Measured dimensions of the ordinary steel columns (mm).

Types	Eccentricities			
	0 mm	10 mm	20 mm	30 mm
Outer diameters	42.23	42.45	42.22	42.36
Thicknesses	5.64	5.57	5.50	5.55
Lengths	2200	2200	2198	2198

**Table 5:** Design initial pretensions.

0.5 *T_opt_*	*T_opt_*	2 *T_opt_*
1.15 kN	2.29 kN	4.58 kN

## Data Availability

Not applicable.
